# The neuroprotective effect of melatonin in glutamate excitotoxicity of R28 cells and mouse retinal ganglion cells

**DOI:** 10.3389/fendo.2022.986131

**Published:** 2022-10-12

**Authors:** Chao Wang, Yaqiong An, Zhaohua Xia, Xuezhi Zhou, Haibo Li, Shuang Song, Lexi Ding, Xiaobo Xia

**Affiliations:** ^1^ Eye Center of Xiangya Hospital, Central South University, Changsha, China; ^2^ Hunan Key Laboratory of Ophthalmology, Central South University, Changsha, China; ^3^ National Clinical Research Center for Geriatric Disorders, Xiangya Hospital, Central South University, Changsha, China; ^4^ Department of Social Medicine and Health Management, Xiangya School of Public Health, Central South University, Changsha, China

**Keywords:** melatonin, glaucoma, nmda, glutamate, oxidative stress, retinal ganglion cell

## Abstract

Glaucoma is the leading cause of irreversible blindness. The progressive degeneration of retinal ganglion cells (RGCs) is the major characteristic of glaucoma. Even though the control of intraocular pressure could delay the loss of RGCs, current clinical treatments cannot protect them directly. The overactivation of N-methyl-D-aspartic acid (NMDA) receptors by excess glutamate (Glu) is among the important mechanisms of RGC death in glaucoma progression. Melatonin (MT) is an indole neuroendocrine hormone mainly secreted by the pineal gland. This study aimed to investigate the therapeutic effect of MT on glutamate excitotoxicity of mouse RGCs and R28 cells. The Glu-induced R28 cell excitotoxicity model and NMDA-induced retinal injury model were established. MT was applied to R28 cells and the vitreous cavity of mice by intravitreal injection. Cell counting kit-8 assay and propidium iodide/Hoechst were performed to evaluate cell viability. Reactive oxygen species and glutathione synthesis assays were used to detect the oxidative stress state of R28 cells. Retina immunofluorescence and hematoxylin and eosin staining were applied to assess RGC counts and retinal structure. Flash visual-evoked potential was performed to evaluate visual function in mice. RNA sequencing of the retina was performed to explore the underlying mechanisms of MT protection. Our results found that MT treatment could successfully protect R28 cells from Glu excitotoxicity and decrease reactive oxygen species. Also, MT rescued RGCs from NMDA-induced injury and protected visual function in mice. This study enriches the indications of MT in the treatment of glaucoma, providing practical research ideas for its comprehensive prevention and treatment.

## Introduction

Glaucoma is the leading cause of irreversible blindness in the world, and it is characterized by progressive degeneration of retinal ganglion cells (RGCs) and their axons, accompanied by visual field defects (1). The global prevalence of glaucoma in the 40- to 80-year-old population is estimated to be 3.5%. With the number and proportion of the elderly population increasing, 111.8 million people are expected to suffer from glaucoma by 2040 (2). At present, the management of glaucoma mainly focuses on the regulation of intraocular pressure (IOP) and slowing its progress (3). Many studies have shown that even controlling the increase in IOP cannot prevent the death of RGCs and progressive visual field defects (4–6). For patients with end-stage glaucoma, there is no effective neuroprotective method. Therefore, seeking effective optic neuroprotective medication for the treatment of glaucoma is necessary.

As an excitatory neurotransmitter, glutamate (Glu) exists widely in retinal neurons and is involved in the signal transmission between photoreceptors, bipolar cells, and RGCs through N-methyl-D-aspartic acid (NMDA) receptors (7, 8). In the pathological state of glaucoma, excess Glu between synapses cannot be effectively removed and can cause NMDA receptor overactivation, calcium overload in nerve cells, and oxidative stress damage, leading to the death of RCGs and degeneration, which is called glutamate excitotoxicity (8). Many studies have confirmed glutamate excitotoxicity to be among the important mechanisms of RGC death in glaucoma progression (9–11).

Melatonin (N-acetyl-5-methoxytryptamine, C_13_N_2_H_16_O_2_) is an indole neuroendocrine hormone mainly secreted by the pineal gland (12). The secretion of MT has a circadian rhythm. After night falls, the synthesize of MT increases, and the secretion level of MT in the body also increases accordingly, reaching a peak at 2-3 am in the morning. The level of MT at night directly affects the quality of sleep. As a classic antioxidant, MT can protect against oxidative stress damage through different mechanisms, including direct scavenging of reactive oxygen species (ROS), regulation of signaling pathways against oxidative stress, and upregulation of glutathione synthesis (GSH). In addition to MT, many of its metabolites also function as ROS scavengers (13). Through different mechanisms, MT can attenuate oxidative stress damage in lipids, proteins, DNA, and many tissues. Besides being secreted from the pineal gland, MT has also been found to be synthesized and released by many ocular structures, including the retina, ciliary body, lens, and Harderian gland in chickens (13, 14). Studies have shown that MT can exert neuroprotective effects through its anti-oxidative stress effect (15–17),, although its role in neuroprotective effects in glaucoma is still unclear.

In this study, we found that MT showed an effective neuroprotective effect against neuronal glutamate toxicity, and it significantly reduced NMDA-induced loss of RGCs. Moreover, MT treatment significantly reversed changes in the retinal transcriptome caused by NMDA. All these results highlight the potential value of MT as a potential medication for neuroprotection treatment in glaucoma.

## Materials and methods

### Cell culture and glutamate excitotoxicity model

Immortalized R28 cells (Key Laboratory of Ophthalmology, Xiangya Hospital, Central South University, Changsha, China) were maintained in low-glucose Dulbecco’s modified Eagle’s medium (11885084, Gibco, Carlsbad, USA) supplemented with 10% fetal bovine serum (FSP500, ExCell Bio, Jiangsu, China) and 1% penicillin-streptomycin (C100C5, NCM Biotech; Zhejiang, China) at 37°C with 5% CO_2_. In the glutamate excitotoxicity model, cells were treated with L-Glutamate (ab120049, Abcam, Cambridge, UK) and incubated for 24 h.

### Cell viability assay

Cell viability was measured by cell counting kit-8 (CCK-8; C6005, NCM Biotech). R28 cells were seeded in 96-well plates at a density of 5000 cells/well and cultured in a medium containing various concentrations of Glu (5, 10, 15, 20, 25 mM). After 24 h incubation, 10% CCK-8 was added and incubated at 37°C for 3h, as per the manufacturer’s instructions. Absorbance was measured at 450 nm using a microplate reader. Meanwhile, propidium iodide (PI)/Hoechst was applied to calculate the R28 cell survival rate after Glu and MT (M5250, Sigma-Aldrich, St. Louis, MO, USA) were treated for 24 h; cells were stained using apoptosis and necrosis assay kit (C1056, Beyotime, Shanghai, China) and pictured using an optical microscope (Eclipse C1, Nikon, Tokyo, Japan).

### ROS assay

Intracellular ROS were detected using a cellular ROS assay kit (ab113851; Abcam). The collected cells were digested with trypsin and then stained in culture media with 20 µM DCFDA and incubated for 30 minutes at 37°C. The cells were washed with 1× buffer after incubation and analyzed immediately with a flow cytometer. Forward and side scatter gates were established to exclude debris and cellular aggregates from the analysis. DCF was excited by the 488 nm laser and detected at 535 nm (typically FL1). The mean florescence intensity (MFI) were analyzed by Flowjo software version 10.0.7.

### Reduced glutathione assay

A micro reduced GSH assay kit (BC1175, Solarbio, Beijing, China) was used to detect reduced GSH. A total of 5 million cells were collected and cleaned twice with PBS. The GSH extract was then added twice for repeated freeze–thaw (frozen in liquid nitrogen and dissolved in a 37°C water bath) and centrifuged at 8000 g for 10 min, and the supernatant was collected at 4°C. The GSH content was detected according to the instructions and standardized according to the number of cells.

### Animals and NMDA-induced retinopathy mouse model

C57BL/6 mice (8 weeks old; Slaccas, Changsha, China) were fed with standard laboratory food and water in a comfortable environment with a 12 h light–dark cycle. All the experimental procedures were approved by the Institutional Animal Care and Use Committee (IACUC) of Central South University (Changsha, China). All mice were divided into Three groups: Sham (only acupuncture without injection), NMDA (20 mM), and MT (20 mM NMDA + 400 mM MT). All the mice were anesthetized with pentobarbital (1%, 80 mg/kg, intraperitoneal injection; Beijing Sanshu, China) and then operated on under a stereomicroscope. Oxybuprocaine hydrochloride (Santen Pharmaceuticals, Tokyo, Japan) was used to induce ocular surface anesthesia, and tropicamide phenylephrine (Santen Pharmaceuticals) was used to dilate the pupils. A 30 G needle was inserted into the vitreous cavity along the limbus and injected at a volume of 1 µL per eye. Tobramycin dexamethasone eye ointment (Alcon Inc, Geneva, Switzerland) was used to prevent infection after injection. The mice were euthanized 5 d after the injection, and their eyeballs were removed with tweezers for the follow-up research.

### Flash visual-evoked potential analysis

Visual function was assessed by flash visual-evoked potential analysis (FVEP) 5 d after intravitreal injection, all after anesthesia. After 15 min of dark adaptation, the following 3 electrodes were fixed separately and inserted under the skin: ground electrode (ack), cathode (anterior bregma), and anode (occipital bone). After covering the contralateral eye, the images of both eyes were measured by a multifocal electroretinography recorder (GT-2008V-VI, Gotec, Chongqing, China) and recorded by Ganzfeld electrodiagnostic system (Gotec). The time of the flash is 100 ms. The first negative wave amplitude and first positive wave latencies were used to assess the visual function in mice.

### Hematoxylin and eosin staining

The mice were euthanized 5 days after modeling, and their eyeballs were removed and fixed with an FAS eyeball fixator (G1109, Servicebio, Wuhan, China). The eyeballs were embedded in paraffin and cut into 4 μm vertical sections. Sections were stained with hematoxylin and eosin (H&E; G1120, Servicebio) according to the manufacturer’s instructions and visualized using an optical microscope (Eclipse C1). CaseViewer software (3DHISTEC, Sysmex, Switzerland) was used to measure the thickness of the ganglion cell layer at distances of 300, 600, 900, 1200, and 1500 μm from the optic nerve center.

### Retina immunofluorescence and RBPMS staining

The mice were euthanized 5 days after modeling, and their eyeballs were removed and fixed with 4% paraformaldehyde (G1101, Servicebio) fixation for 1 h, and the retinas were detached under a stereomicroscope. The retinas were sealed with 5% bovine serum albumin and 0.5% Triton-X100 in PBS for 1.5 h at room temperature, followed by incubation with primary antibody RBPMS (ab152101, Abcam) at 4°C overnight. They was cleaned with 0.5% Triton-X100 in PBS 4 times for 5 min each were then incubated with fluorescent-labeled secondary antibody away from light for 2 h at ambient temperature. The retinas were then viewed and pictured by optical microscope (Eclipse C1).

### RNA sequencing

The mouse retinas were collected 5 days after NMDA intervention. Three individual retinas were treated as one sample, and each group contained 3 samples. RNA was isolated by total RNA kit (R6834-01, Omega Bio-Tek),. Total amounts and integrity of RNA were assessed using the RNA Nano 6000 assay kit of the Bioanalyzer 2100 system (Agilent Technologies, CA, USA). After RNA was converted to cDNA, the samples were sequenced by the Illumina NovaSeq 6000 at Novogene (Beijing, China). Genes with a fold-change ≥1.5 identified by edgeR and a false discovery rate <0.05 were considered differentially expressed (BMKCloud, http://www.biocloud.net/). Gene functional annotations were based on the Kyoto Encyclopedia of Genes and Genomes (KEGG, https://www.genome.jp/kegg/) and Gene Ontology (GO, http://www.geneontology.org/) databases.

### Statistical analysis

SPSS 26.0 statistical software (IBM Corp., Armonk, NY, USA) was used for statistical analysis of all data. All data were presented as the mean ± standard deviation (SD). One-way analysis of variance (ANOVA) was used to assess the significance differences of cell viability, ROS, GSH, RGCs survival and FVEP results between groups. Repeated measures ANOVA was used to assess thickness of retinal ganglion cell complex (GCC). Charts were built using GraphPad Prism 6.0 (GraphPad Inc., La Jolla, CA, USA). P value <0.05 was statistically significant.

## Results

### MT protects R28 cells from Glu-induced excitotoxicity

**Figure 1:**
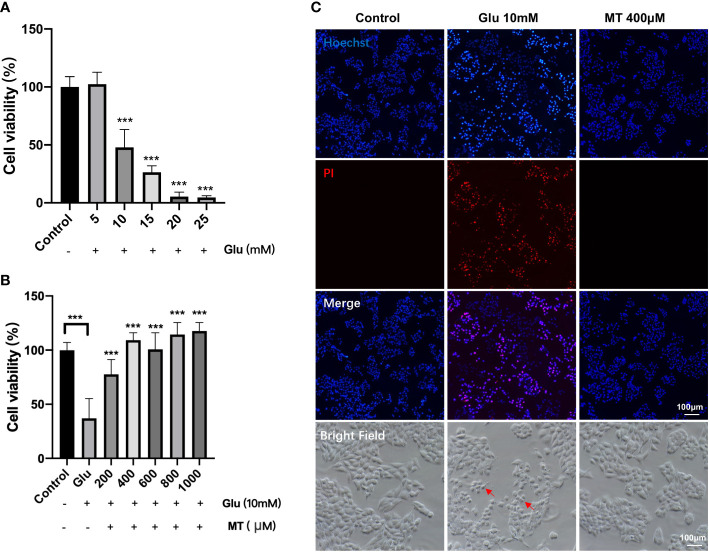
MT protects R28 cells from glutamate-induced excitotoxicity. **(A)** Effects of different concentrations of Glu on R28 cell viability after 24 h treatment. (n = 4) **(B)** Protective effect of different concentrations of MT on R28 cells treated with 10 mM glutamate for 24h. (n=6) **(C)** Pictures of R28 cells stained by PI and Hoechst. Red arrows: glutamate-induced excitotoxicity of R28 cells. Data are the mean ± SD; ***p < 0.001. Scale bar = 50 µm.

To investigate the appropriate concentration of Glu, R28 cells were treated with 5-25mM Glu at different concentrations for 24 h. CCK-8 assay results showed that cell viability decreased gradually with increasing Glu concentration in a concentration-dependent manner. Compared with the control group, 10 mM (47.90 ± 15.50%), 15 mM (26.41 ± 5.48%), 20 mM (5.41 ± 3.86%), 25 mM (4.73 ± 1.43%) significant decreased cell viability with Glu treatment for 24 h. (P <0.001, n = 4) (**Figure 1A**). In subsequent experiments, R28 cells were treated with 10 mM Glu for 24 h as the immobilization condition. Subsequently, we investigated the protective effect of different concentrations of MT on glutamate-induced excitotoxicity injury. The results showed that compared with the Glu group, the cell viability of the MT group was significantly increased with the increasing MT concentration. The cell viability reached 109.1 ± 6.9% when the concentration of MT was at 400 μM (P <0.001, n = 6) (**Figure 1B**), suggesting that MT has a protective effect on Glu-induced R28 cell damage, and the MT at concentration of 600 μM, 800 μM and 1000 μM also showed good protective effect (P <0.001, n = 6). Meanwhile, PI/Hoechst staining was used to confirm this view, and it was observed that R28 cells died more after 24 h of glutamate treatment, while MT saved this damage (**Figure 1C**). These results suggest that MT can protect R28 cells from glutamate-induced excitotoxicity.

### MT protects R28 cells from Glu-induced oxidative stress

**Figure 2:**
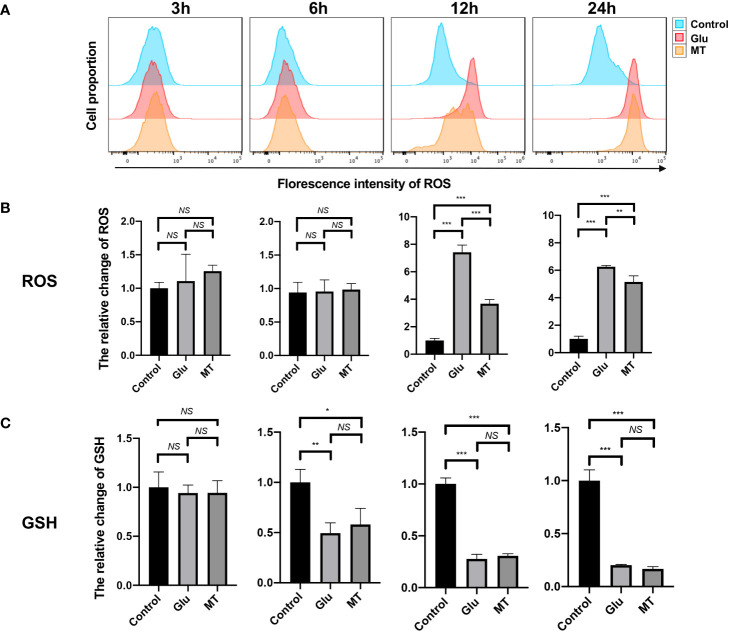
MT protects R28 cells from Glu-induced oxidative stress. **(A, B)** ROS levels increased gradually over time after glutamate treatment, and MT could ameliorate this change. (n = 3) **(C)** GSH levels decreased over time, but MT treatment could not rescue this change. (n = 3) Data are the mean ± SD; NS, No Significant, *p < 0.05, **p < 0.01, ***p < 0.001.

To investigate the effects of MT on Glu-induced oxidative stress in R28 cells, intracellular ROS and reduced GSH levels were detected. The results showed that ROS levels increased gradually over time after Glu treatment and peaked at 12 h (7.42 ± 0.52), and MT treatment could ameliorate the changes induced by Glu (3.67 ± 0.30, P <0.001, n = 3) (**Figures 2A, B**). At 24 h, MT was still protective (5.14 ± 0.43, P <0.01, n = 3), but this was not as significant as at 12 h (**Figures 2A, B**). Meanwhile, with the increase in Glu treatment time, the intracellular GSH level gradually decreased and reached its lowest at 24 h (0.20 ± 0.01, P <0.001, n = 3) (**Figure 2C)**. However, the GSH level of the MT group was not significantly improved when compared with the Glu group (0.16 ± 0.02, P >0.05, n = 3) (**Figure 2C**).

### MT protects against NMDA-induced retinal damage in mice

**Figure 3:**
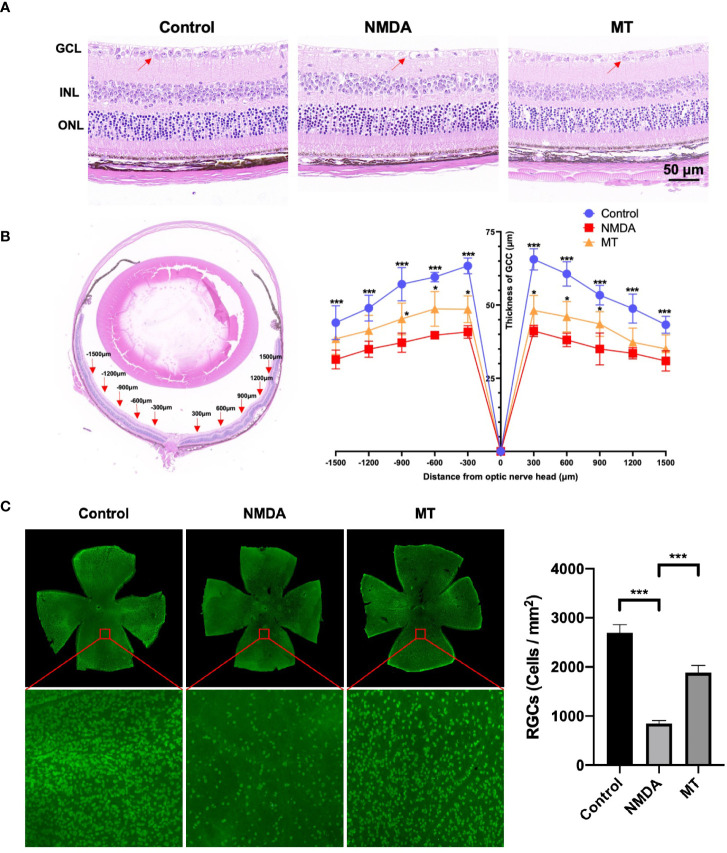
MT protects against NMDA-induced retinal damage in mice. **(A)** Images of H&E staining sections of mice retina at 5 days after intravitreal injection (Scale bar = 50 μm). Red arrows: RGCs. **(B)** At 5 days after intravitreal injection of NMDA, the GCC thickness of mice was measured ±300, ± 600, ± 900, ±1200, and ±1500 μm away from the optic nerve. (n=4) **(C)** Labeling of RGCs with RBPMS 5 days after intravitreal injection of NMDA. MT improved the density reduction of NMDA-induced RGC injury in mice (Scale bar = 100 μm). (n=4) Data are the mean ± SD; *p < 0.05, ***p < 0.001.

To further determine the protective effect of MT on retinal excitotoxicity, the thickness of GCC was measured after H&E staining, and RGCs were counted and quantitatively analyzed after being labeled with RBPMS by retinal immunofluorescence. H&E staining showed that the retinal GCC thickness of mice in the NMDA group was significantly thinner than that in the control group 5 days after intravitreal injection of NMDA (P <0.001, n = 4) (**Figures 3A, B**). MT treatment could effectively inhibit the thinning of the GCC layer caused by NMDA at 300, 600 and 900 μm from the optic nerve center (P <0.05, n = 4). Retinal immunofluorescence showed that the density of RGCs in the NMDA group was significantly lower than that in the control group, while the number of surviving RGCs in the MT group (1883.10 ± 124.63) was significantly better than that in the NMDA group (849.30 ± 47.10) but still lower than that in the control group (2694.60 ± 145.85, P <0.001, n = 4) (**Figure 3C**). These results suggest that MT has a protective effect on NMDA-induced retinal injury in mice.

### MT protects visual function in mice

**Figure 4:**
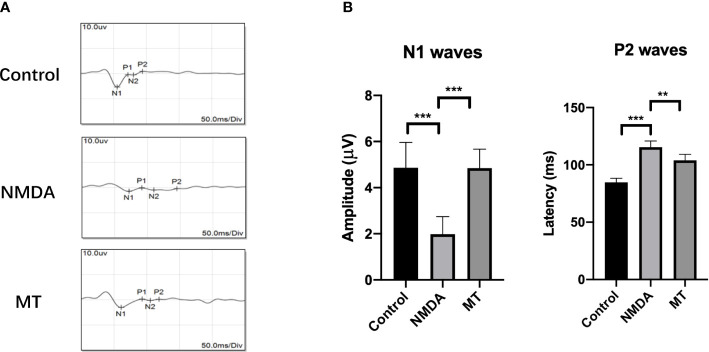
MT protects visual function in mice. **(A)** FVEP images of mice after intravitreal injection of NMDA 5 days. **(B)** The amplitude of N1 waves and the latencies of P2 waves of FVEPs in mice at 5 days after injection. (n = 6) Data are the mean ± SD; **p < 0.01, ***p < 0.001. Scale bar = 10.0 μV and 50 ms.

We also studied the effect of MT on electrophysiological activity of the retina and its protective effect on visual function in mice. The amplitude of N1 wave was decreased NMDA treatment (1.98 ± 0.76 µV) compared with control group (4.87 ± 1.10 µV), and MT increase amplitude of N1(4.85 ± 0.82 µV, P <0.001, n = 6).The latencies of P2 wave were prolonged 5 days after intravitreal injection in the NMDA group (115.42 ± 5.45 ms) compared with control group (84.83 ± 3.52 ms), and MT ameliorated this change (103.91 ± 5.28, P <0.005, n = 6) (**Figure 4**). These results suggest that NMDA causes retinal dysfunction in mice, and MT can improve the visual conduction dysfunction induced by excitotoxicity.

### MT ameliorated transcriptome abnormalities in NMDA-induced retinal injury

**Figure 5:**
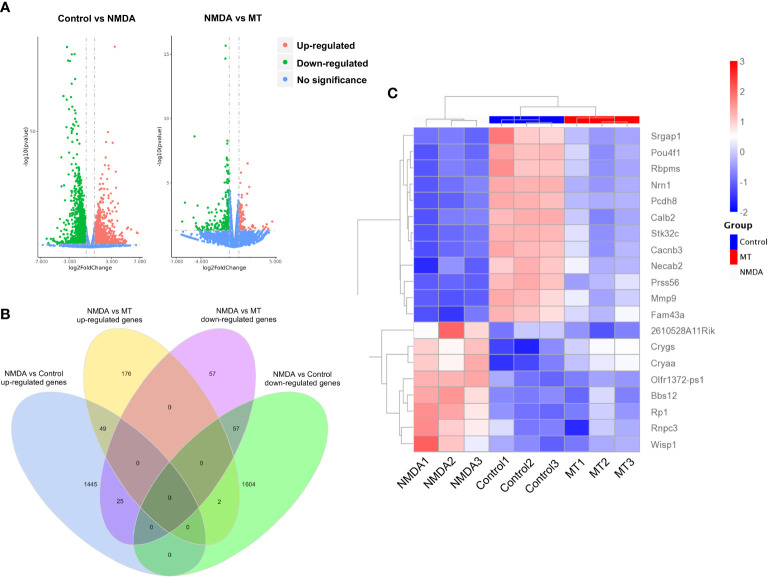
MT ameliorates retinal transcriptome abnormalities in NMDA-induced retinal injury. **(A)** Volcano plot of differentially expressed genes between control, NMDA, and MT groups. **(B)** Venn diagram shows the differentially expressed genes among the control, NMDA, and MT group. **(C)** The heat map shows MT-restored top 20 genes.

To investigate further the mechanism of the neuroprotective effects of MT on the retina, RNA sequencing analysis was performed. Compared with the control group, the NMDA-treated group had 1519 upregulated genes and 1663 downregulated genes. With the intervention of the MT, 139 genes were upregulated and 227 genes downregulated (**Figure 5A**). MT treatment mitigated the expression of approximately 49 upregulated genes and 57 downregulated genes induced by NMDA (**Figure 5B**). These genes included 5 neuroactive ligand-receptor interaction-related genes (Oprl1/Ptafr/Adcyap1r1/Lpar6/Crhr1), 3 PI3K-Akt signaling pathway-related genes (Col6a3/Lpar6/Gng4), and 3 calcium signaling pathway-related genes (Ptafr/Prkcg/Orai3). These results suggest that MT exhibits its neuroprotective effect by ameliorating retinal transcriptome abnormalities (**Figure 5C**).

### Analysis of differentially expressed genes in NMDA-treated and MT-treated mice retinas

**Figure 6:**
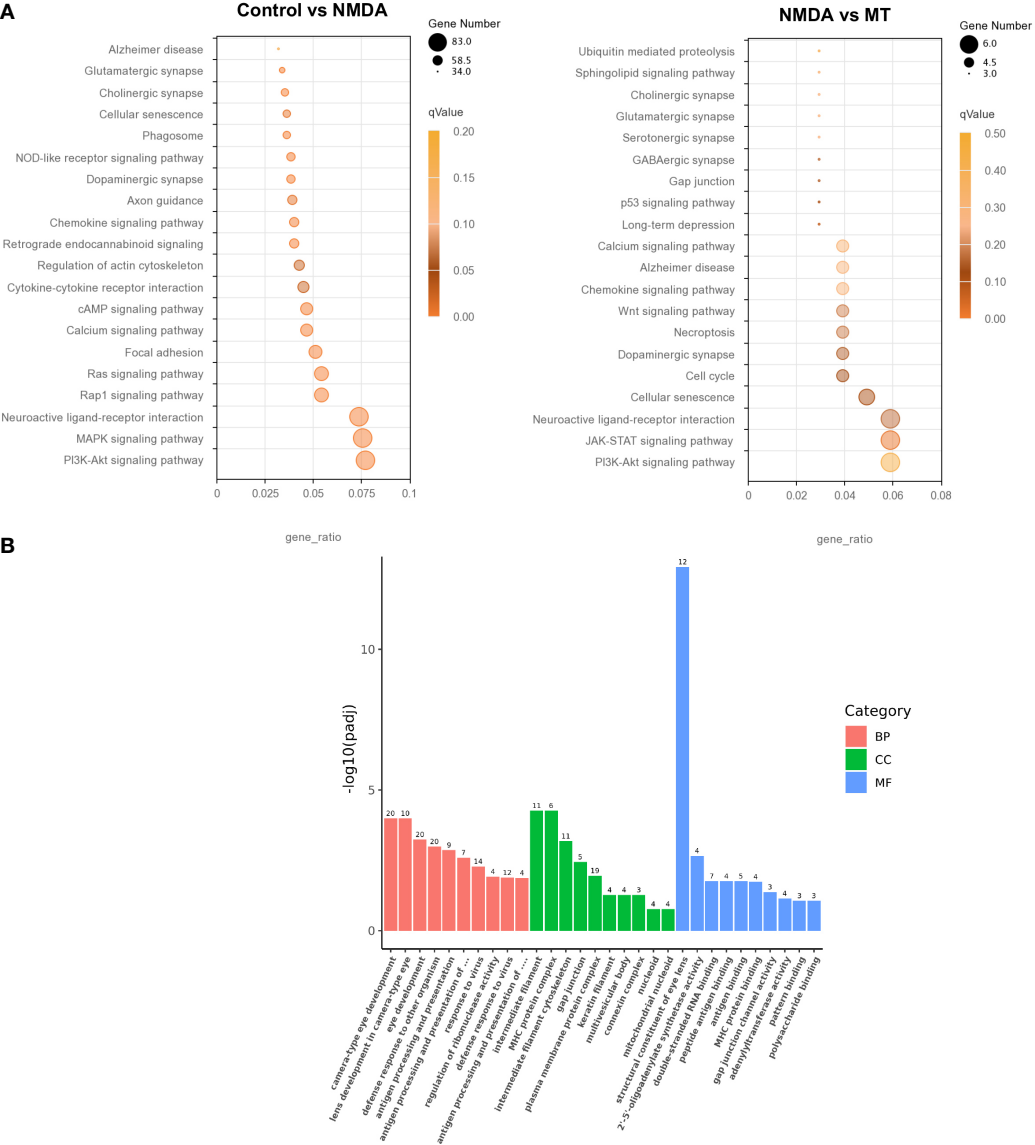
Analysis of differentially expressed genes in NMDA- and MT-treated mice retina. **(A)** KEGG analysis of differentially expressed genes between control, NMDA, and MT group. **(B)** Go analysis of differentially expressed genes between control, NMDA, and MT group in biological process (BP), cellular component (CC), and molecular function (MF).

KEGG and GO analyses were performed to densify the signaling pathway and biological process changes in the retina. We performed KEGG analysis on the differentially expressed genes in the control, NMDA, and MT groups. This indicated that the PI3K-Akt and MAPK signaling pathways were both crucial after NMDA intervention. After MT treatment, the PI3K-Akt and JAK-STAT pathways were involved in rescuing the injury induced by NMDA (**Figure 6A**). GO analysis results showed that the retinal biological process, cellular component, and molecular function were all altered by MT intervention. The differentially expressed genes were enriched in the biological process (**Figure 6B**).

## Discussion

The characteristic death of RGCs is one of the most important features of glaucoma, which can cause irreversible visual field defects and seriously affect the life quality of patients (18, 19). Although many glaucoma medications have been applied in clinical treatment, their use for glaucomatous neuroprotection is still very limited, and there are no clear clinical outcomes (20, 21).

Many studies have shown a potential relationship between MT and glaucoma. Patients with glaucoma are often accompanied by sleep disturbances, anxiety, and depression, and studies have shown that glaucoma is also associated with disturbances in the rhythm of MT secretion (22, 23). Recent studies showed that urinary 6-sulfatoxymelatonin, the main metabolite of serum MT in glaucoma patients, is significantly lower than normal, suggesting the possibility of a circadian rhythm disturbance in glaucoma patients, which MT can restore (24).

Focusing on the eye, although MT can be secreted by various eye structures, and the aqueous humor also contains a certain concentration of MT (25), the role of MT in the eye is still unclear. Concentrations of MT have been shown to be 3 times higher in aqueous humor in patients with elevated IOP than in normal patients, and the same was also observed in a mouse model of glaucoma (26). Preoperative treatment with oral MT has been shown to reduce IOP in patients who have undergone cataract surgery (27). Animal models and clinical trials have also shown that MT and its analogs can reduce IOP (28, 29). Some studies have demonstrated that MT exerts antiapoptotic and neuroprotective effects on retinal neurons after hypoxia-ischemia and acute intraocular hypertension (30, 31). In our study, we established the classical NMDA-induced retinal injury model, which imitated the different mechanisms of RGC death in the pathogenesis of glaucoma. We found that MT has a significant protective effect on cellular Glu excitotoxicity both *in vivo* and *in vitro* and provides a supplement to the protective role of melatonin in the pathogenesis of different glaucoma.

We found that MT at a concentration of 400 μM had a 100% protective effect on Glu-induced cell excitotoxicity in R28 cells, and a high concentration of 1000 μM had no toxic effect on cells, confirming that MT is effective and safe. MT also showed a good neuroprotective effect *in vivo*. MT rescued NMDA-induced RGC loss and GCC thinning, and through the detection of FVEP, it was confirmed that melatonin can restore partial visual function in mice. As an endocrine hormone with strong anti-oxidative stress ability, MT has strong potential for the neuroprotection of glaucoma.

As a classic antioxidant, MT can effectively scavenge ROS and increase the content of intracellular GSH to resist oxidative stress injury (32). In our study, we found that after glutamate excitotoxicity injury, although MT had a significant protective effect on R28 and RGCs and a significant recovery of visual function in mice, it did not increase the content of GSH. As a common antioxidative product, its depletion is much greater than its synthesis in the glutamate excitotoxicity process. ROS was significantly higher than that in the Glu group at 24 h, but it still had a 5.16-fold increase compared to the control group. Therefore, we speculate that, in addition to the scavenging effect of MT on ROS, other mechanisms also play a key role in the process of neuroprotective effect.

To explore the mechanism of action of the neuroprotective effect of MT, we conducted RNA sequencing of the retina, and the sequencing results of this study found that MT rescued abnormal retinal transcriptome expression induced by NMDA. Through further gene enrichment, we found significant changes in the PI3K-AKT and MAPK signaling pathways in NMDA-induced retinal injury, and after MT treatment, different genes were enriched to the P13K-AKT and JAK-STAT signaling pathways. This is consistent with many of the studies that showed PI3K-AKT and MAPK to be involved in the occurrence and development of glaucoma and play an important role in the death of RGCs (33–36). Studies have also shown that RGCs are protected by intervening PI3K-AKT and JAK-STAT signaling pathways through regulating apoptosis, autophagy, and oxidative stress processes (37–41). In our study, sequencing results showed a neuroprotective effect, suggesting that MT may depend on the above pathways. However, the specific mechanism of action needs to be studied further.

## Conclusion

This study explored the neuroprotective effects of MT on NMDA-reduced RGC death and Glu-induced R28 cell excitotoxicity. It found that MT successfully rescues RGCs from NMDA-reduced injury and protects visual function in mice. MT also protects R28 cells from Glu excitotoxicity and decreases ROS. RNA sequencing indicated that MT treatment repairs the abnormal transcriptome caused by NMDA, and PI3K-AKT and JAK-STAT signaling pathways may play an important role in this process. This study provides practical research ideas for the comprehensive prevention and treatment of glaucoma.

## Data availability statement

The datasets presented in this study can be found in online repositories. The names of the repository/repositories and accession number(s) can be found here: https://www.ncbi.nlm.nih.gov/bioproject/, SRA PRJNA867307.

## Ethics statement

The animal study was reviewed and approved by Institutional Animal Care and Use Committee (IACUC) of Central South University.

## Author contributions

CW and YA wrote the first draft of the paper. XX and LD edited the paper. XX, LD and CW designed research. CW, YA performed laboratory research. ZX performed FVEP examination. XZ performed bioinformatics Analysis, CW, YA and SS analyzed data. All authors contributed to the article and approved the submitted version.

## Funding

This study was financially supported by The National Key Research and Development Program of China (No.2020YFC2008205), The National Natural Science Foundation of China (No. 82171058, No.81974134 for XX, No.82070966 to LD, No.8210041510 to CW), Key R&D Plan of Hunan Province of China (No.2020SK2076 to XX), Science and Technology Innovation Program of Hunan Province (No.2021RC3026 to LD), Natural Science Foundation of Hunan Province (No.2021JJ41021 to CW) and China Postdoctoral Science Foundation (No.2021M693556 to CW).

## Acknowledgments

We thank Lemeng Feng, Weizhou Fang, Shirui Dai, Cheng Zhang and Wulong Zhang for technical assistance. We thank Scribendi (https://www.scribendi.com/) for editing the English text of a draft of this manuscript.

## Conflict of interest

The authors declare that the research was conducted in the absence of any commercial or financial relationships that could be construed as a potential conflict of interest.

## Publisher’s note

All claims expressed in this article are solely those of the authors and do not necessarily represent those of their affiliated organizations, or those of the publisher, the editors and the reviewers. Any product that may be evaluated in this article, or claim that may be made by its manufacturer, is not guaranteed or endorsed by the publisher.
